# P16INK4a upregulation mediated by TBK1 induces retinal ganglion cell senescence in ischemic injury

**DOI:** 10.1038/cddis.2017.169

**Published:** 2017-04-20

**Authors:** L u Li, Yin Zhao, Hong Zhang

**Affiliations:** 1 Department of Ophthalmology, Tongji Hospital, Tongji Medical College, Huazhong University of Science and Technology, Wuhan, China; 2 Department of Ophthalmology, The First Affiliated Hospital, Shihezi University School of Medicine, Shihezi, Xinjiang, People's Republic of China

## Abstract

Glaucoma is a leading cause of irreversible blindness worldwide that is characterized by progressive retinal ganglion cell (RGC) death. However, RGC senescence as a phase before RGC death, and the mechanism of RGC senescence remains unclear. Here, we demonstrate that TANK-binding protein 1 (TBK1) is upregulated in acute IOP elevation-induced ischemic retinas mouse model. Moreover, pre-treatment with the TBK1 inhibitor BX-795 reduced p16INK4a (p16) expression and RGC senescence. Upregulation of TBK1 via plasmid transfection increased Akt phosphorylation at Ser473 and Bmi1 phosphorylation. The Akt inhibitor MK-2206 decreased the expression of p16 and Bmi1 serine phosphorylation. A Bmi1 Ser316 mutation also attenuated TBK1-induced p16 upregulation. Finally, silencing of TBK1 via shRNA knockdown reduced the expression of p16 as well as Akt and Bmi1 phosphorylation, reducing RGC senescence *in vivo*. These data suggest that acute IOP elevation-induced ischemia increases TBK1 expression, which then increases p16 expression through the Akt- Bmi1 phosphorylation pathway. This study therefore elucidates a novel mechanism whereby TBK1 regulates p16 expression and RGC senescence, suggesting a potential novel treatment strategy for minimizing RGC senescence in retinal ischemia and glaucoma.

Glaucoma is the leading cause of permanent vision loss and irreversible blindness worldwide and is characterized by accelerated and progressive retinal ganglion cell (RGC) death, axonal degeneration and optic nerve excavation.^1, 2^ Elevated intraocular pressure (IOP), advanced age and genetics are considered to be the main risk factors for disease onset. Although the underlying molecular mechanisms of RGC death in glaucoma have not been completely clarified, recent evidence has demonstrated that cellular senescence can cause elevated rates of RGC death and subsequent blindness.^3, 4^ Therefore, studies on the mechanisms of RGC senescence may aid in understanding the pathogenesis of glaucoma and developing new therapeutic interventions.

Genetic research breakthroughs have indicated that the *TBK1* and *p16INK4a (p16)* loci are among those most strongly associated with glaucoma.^5, 6^ TANK-binding kinase 1 (TBK1) is a serine-threonine kinase belonging to the non-canonical IκB kinases, which are involved in the activation of various cellular pathways, including NF-κB and IRF3, and whose target genes include type I and type III interferons.^7^ TBK1 is as an essential player in autophagy and phosphorylation of the autophagic adaptor p62.^8^ Human partial *TBK1* deficiency results in neuroinflammatory and neurodegenerative disorders of the central nervous system, such as childhood herpes simplex encephalitis and amyotrophic lateral sclerosis. The *TBK1* mutation results in normal tension glaucoma.^9^ Therefore, TBK1 acts as a key regulator of neuroinflammation, immunity and autophagy and may be involved in the pathogenesis of glaucoma. Although initial studies have shown that TBK1 is expressed in human RGCs and in tissues affected by glaucoma, the role of TBK1 in RGCs and in the pathology of RGC senescence in glaucoma remains an unanswered question.

The senescence marker p16INK4a (cyclin-dependent kinase inhibitor 2A, isoform INK4a) is an indicator of irreversible growth arrest (senescence) in cultured cells and tissues. Selective elimination of p16-positive cells can prevent or delay senescent deterioration.^10, 11^ A recent study has further confirmed that *p16* mRNA expression levels are dramatically increased in the retinas of acute IOP-elevated mouse, suggesting the involvement of a p16-dependent RGC senescence pathway in the progression of glaucoma. The expression of p16 was found to be significantly elevated in a rat model of glaucoma, also, delineating a time point corresponding to ongoing RGC death.^4^ In addition, the cell cycle proteins cyclin A is expressed in reversibly damaged cells *in vivo* after transient focal ischemia, which regulate entry into and progression through S phase.^12^ RGC senescence as a phase before RGC death may refer to the expression of cyclin A, while RGCs are typically described as terminally differentiated.

The Polycomb group protein Bmi1 is a critical regulator of cellular senescence, playing a key role in inhibition of the Ink4a/Arf locus and affecting the abundance of p16.^13^ Bmi1-deficient primary mouse embryonic fibroblasts are impaired in progression into the S phase of the cell cycle and undergo premature senescence.^14^ In addition, Bmi1 can be phosphorylated at different serine residues, modulating its function through the Ink/Arf pathway beyond the cellular level.^15^ A consensus Akt phosphorylation site in Bmi1 Ser316 is conserved across species. Re-expression of Bmi1 S316A phosphorylation in Bmi1^−/−^ cells prevents premature senescence, while re-expression of Bmi1 S316D phosphorylation can upregulate the expression of P16, indicating that phosphorylation of Bmi1 S316D impairs the function of Bmi1.^16^ Moreover, Akt-mediated phosphorylation of Bmi1 also inhibits its ability to promote self-renewal of hematopoietic stem and progenitor cells. These studies indicate that the activation of Akt regulates Bmi1 and that Akt phosphorylation of Bmi1 at Ser316 impairs its function.

Akt is serine/threonine kinase that acts as a direct effector, TBK1 acts to promote Akt activation through direct stimulation of Akt catalytic activity via TBK1-induced phosphorylation of both the canonical activation loop (T308) and hydrophobic motif (S473).^17^ In addition, both the PI3K-Akt pathway and Bmi1 are vital to cell self-renewal, metabolism, migration and senescence, acute activation of the pathway in normal stem cells can lead to senescence.^18, 19^ Although the TBK1, Akt, Bmi1 and p16 correlate to cellular senescence, however, the precise mechanisms by which acute IOP elevation-induced ischemic retinas lead to RGC senescence are not well understood.

As a well-known animal model, the acute IOP elevation-induced ischemic injury has been used to study RGC damage and elaborate the possible molecular mechanism of injured cells.^3, 20, 21^ In the present study, we investigated the components of the signalling pathway that mediates RGC senescence and identified the key players that interact with TBK1 and p16. We found that acute IOP elevation-induced retinal ischemia directly increases TBK1 expression, triggering Akt S473 phosphorylation, activating Bmi1 phosphorylation, and upregulating the expression of p16, which leads to RGC senescence in cell culture and an IOP-induced retinal ischemia mouse model. These findings provide insight into the mechanism of RGC senescence in glaucoma and suggest potential therapeutic targets for halting RGC senescence and reducing damage caused by elevated IOP.

## Results

### TBK1 and p16 are upregulated during RGC senescence in acute IOP elevation-induced ischemia mouse model

**Figure 1:**
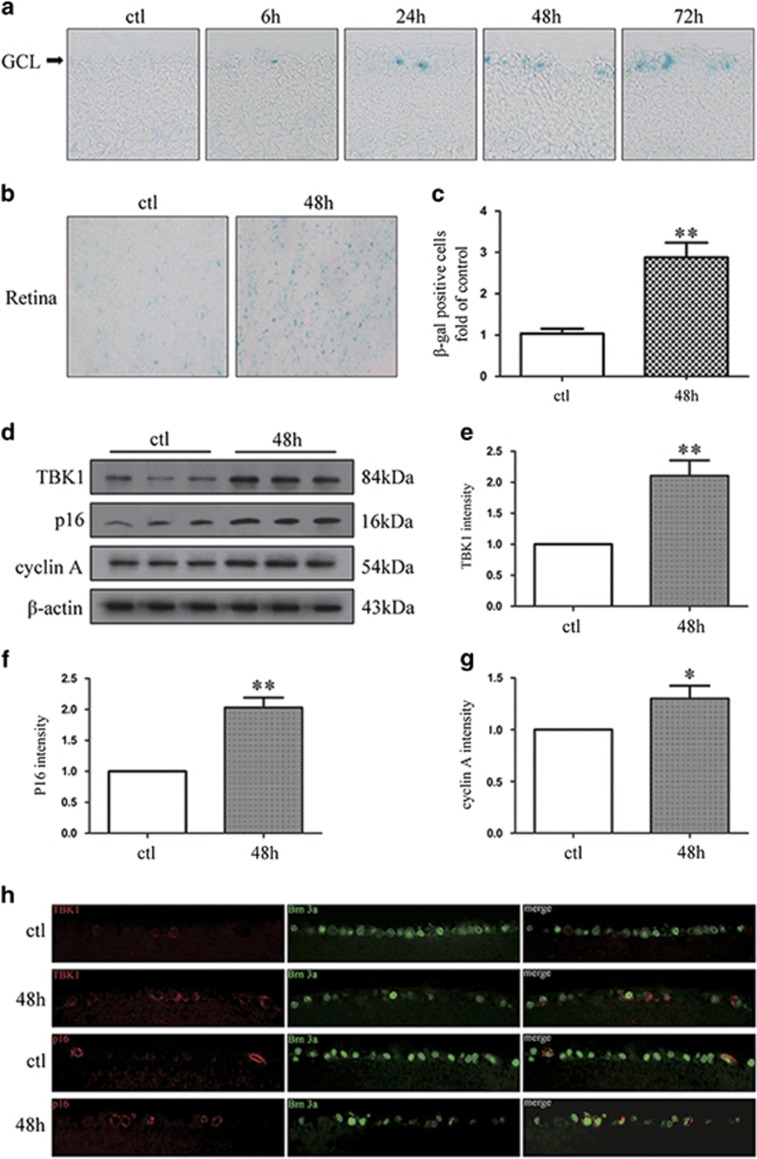
The expression of TBK1 and RGC senescence after acute IOP elevation-induced ischemic retinas *in vivo*. (**a**) Representative *β*-gal staining images of frozen retinal slice after acute IOP elevation-induced ischemic injury. (**b**) Representative *β*-gal staining images of retina flat after IOP elevation-induced ischemic injury. (**c**) Statistical analysis of the data shown in (**b**). The data are expressed as the means±s.e.m. from three independent experiments. **P<0.01 versus control. (**d**) Western blot showing TBK1, p16 and cyclin A expression in ischemic retina. (**e**-**g**) Statistical analysis of the data shown in (**d**). The data are expressed as the means±s.e.m. from three independent experiments. *P<0.05 versus control and **P<0.01 versus control. (**h**) Representative images of TBK1 and p16 expression in ischemic retina slice

To investigate RGC senescence, we used an acute IOP elevation-induced ischemia mouse model that involved elevating the IOP to 75 mm Hg for 45 min, and performed senescence-associated *ββ*-galactosidase (SA-*β*-gal) staining at 6, 24, 48 or 72 h after acute elevation of IOP. As shown in Figure 1a, SA-*β*-gal staining in frozen retinal sections revealed that RGC senescence was initiated 6 h after reperfusion and peaked at 48 h. The number of senescent retinal cells was significantly different between the control group and the 48-h group (Figures 1b and c). Additionally, we examined the expression of TBK1 and p16 following IOP elevation-induced retinal ischemia. Immunofluorescence analysis demonstrated that expression levels of TBK1 and p16 were higher in the 48-h group than control (Figure 1h), which was confirmed by western blotting (Figures 1d−g). These results indicated the pivotal role of TBK1 and p16 in mediating retinal ischemic damage and RGC senescence.

### TBK1 inhibitor BX-795 decreases RGC senescence and p16 expression *in vivo*

**Figure 2:**
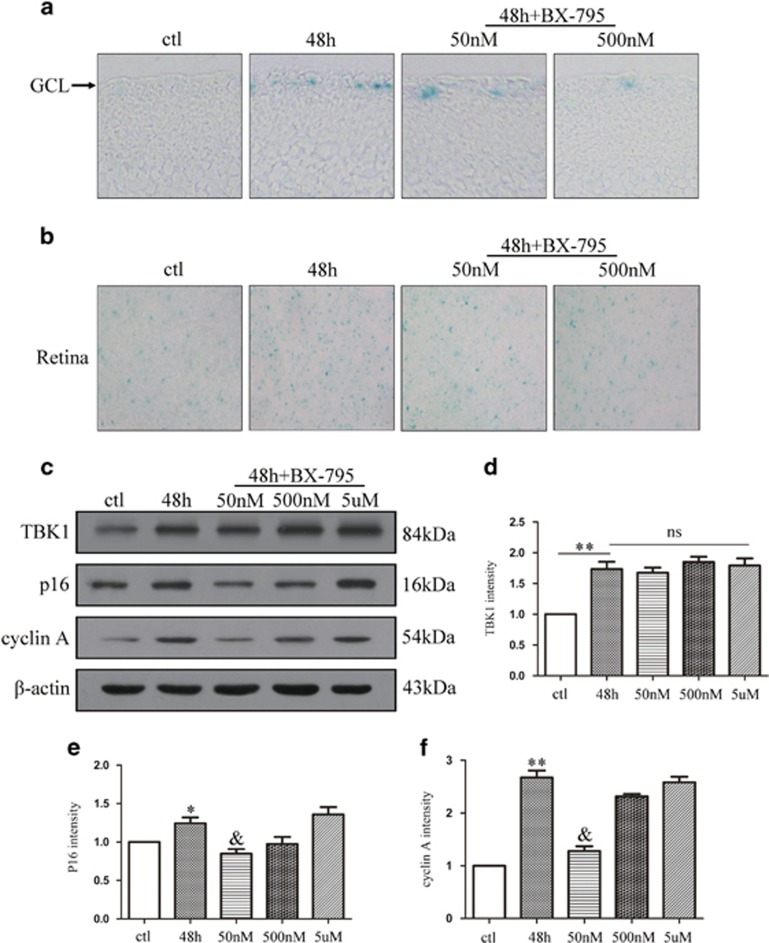
The effect of BX-795 on p16 expression and RGC senescence after acute IOP elevation-induced ischemic retinas *in vivo*. (**a**) Representative *β*-gal staining images of retina slice after acute IOP elevation-induced ischemic retinas. (**b**) Representative *β*-gal staining images of retina flat after IOP elevation-induced ischemic injury. (**c**) Western blot showing the effect of TBK1 inhibitor BX-795 on TBK1, p16 and cyclin A expression in ischemic retina. (**d**-**f**) Statistical analysis of the data shown in (**c**).The data are expressed as the means±s.e.m. from three independent experiments. *P<0.05 versus control, **P<0.01 versus control and ^&^*P*<0.05 versus 48 h group

To confirm that TBK1 is involved in RGC senescence caused by acute IOP elevation-induced retinal ischemia, the TBK1 inhibitor BX-795 was injected into the vitreous in concentrations of 50 nM, 500 nM or 5 *μ*M. We performed western blot analysis to determine protein levels of TBK1, p16 and cyclin A, as well as SA-*β*-gal staining of frozen retinal sections and retinal flats. As shown in Figures 2a and b, retinal ischemia damage increased RGCs' senescence in frozen retinal sections and the number of senescent cells in retinal flats; by contrast, the inhibition of TBK1 reduced the retinal ischemic damage. Expression of TBK1 did not differ between 48-h group and 48-h+BX-795 intravitreal injection, while protein levels of p16 and cyclin A were significantly reduced in ischemia retinal tissue with 50 nM BX-795 intravitreal injection (Figures 2c–f). These findings suggested that TBK1 is involved in RGC senescence and inhibition of TBK1 activation reduces RGC senescence in acute IOP elevation-induced retinal ischemia.

### TBK1 overexpression promotes cellular senescence and p16 upregulation *in vitro*

**Figure 3:**
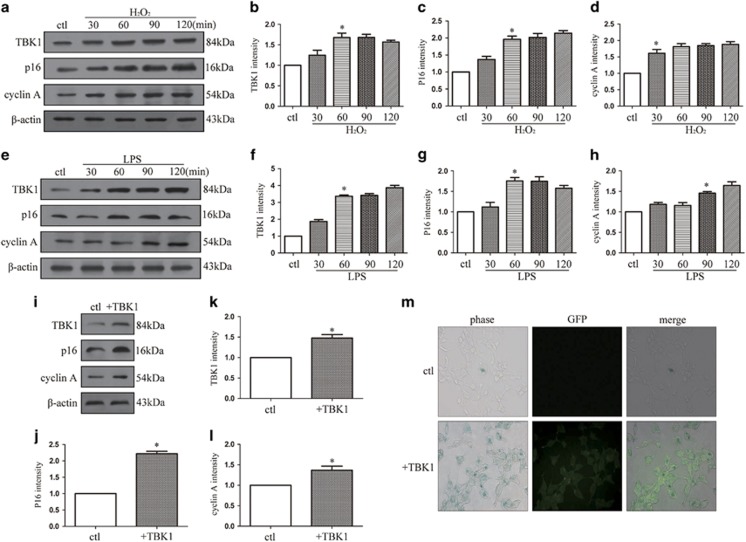
The effect of TBK1 overexpression on p16 expression and cell senescence *in vitro*. (**a**) Western blot showing the expression of TBK1, p16 and cyclin A after H_2_O_2_ (100 *μ*M) stimulation in HEK293. (**b**-**d**) Statistical analysis of the data shown in (**a**). The data are expressed as the means±s.e.m. from three independent experiments, *P<0.05 versus control. (**e**) Western blot showing the expression of TBK1, p16 and cyclin A after LPS stimulation in HEK293. (**f**-**h**) Statistical analysis of the data shown in (**e**).The data are expressed as the means±s.e.m. from three independent experiments, *P<0.05 versus control. (**i**) Western blot showing the effect of TBK1 overexpression on TBK1, p16 and cyclin A expression in HEK293. (**j**-**l**) Statistical analysis of the data shown in (**i**).The data are expressed as the means±s.e.m. from three independent experiments, *P<0.05 versus control. (**m**) Representative *β*-gal staining images showing the effect of TBK1 overexpression on HEK293 cell senescence

To explore the association between RGC senescence and expression levels of TBK1 and p16, HEK293 cells were stimulated by H_2_O_2_ (100 *μ*M) and LPS (1 *μ*g/ml) to imitate cell injury during retinal ischemic injury. After induction of apoptosis in HEK293 cells by H_2_O_2_ for 60 min, expression levels of TBK1, p16 and cyclin A were significantly elevated (Figures 3a–d). Similarly, when inflammation was initiated in HEK293 cells through stimulation with 1 *μ*g/ml of LPS, TBK1, p16 and cyclin A protein levels were upregulated (Figures 3e–h). To further elucidate the relationships among TBK1, cellular senescence and p16, a plasmid containing full-length TBK1 was transfected into HEK293 cells. Western blot results revealed that overexpression of TBK1 led to an increase in levels of p16 and cyclin A (Figures 3i–l). As shown in Figure 3m, transfection of TBK1 was observed by immunofluorescence, and SA-*β*-gal staining was used to label senescent cells. Following transfection with TBK1 plasmid, the number of senescent HEK293 cells increased considerably compared with those in the control group. Together, these results suggest a pivotal role of TBK1 in promoting cellular senescence and p16 expression.

### TBK1 inhibitor BX-795 decreases cellular senescence and p16 expression *in vitro*

**Figure 4:**
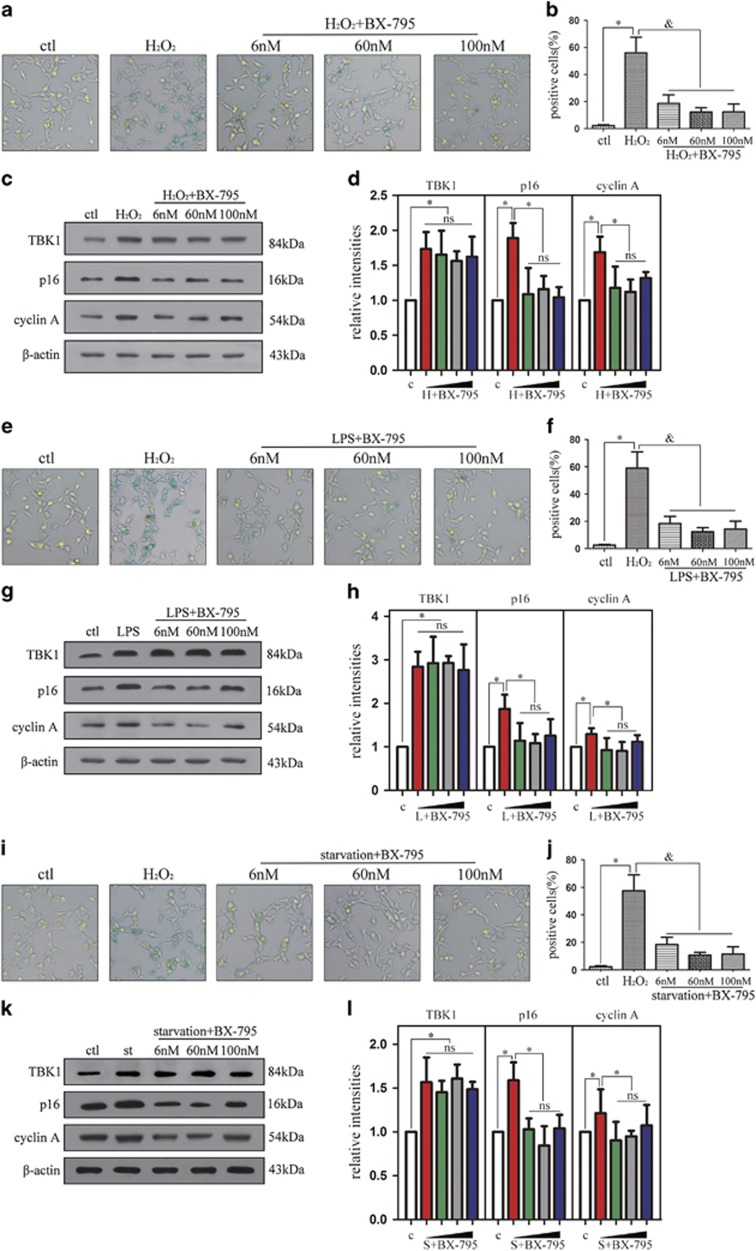
The effect of BX-795 on p16 expression and cell senescence *in vitro*. (a) Representative *β*-gal staining images showing the effect of BX-795 on cell senescence after H_2_O_2_ (100 *μ*M) stimulation 60 min in HEK293. (**b**) Statistical analysis of the data shown in (**a**).The data are expressed as the means±s.e.m.. from three independent experiments, *P<0.05 versus control, ^&^*P*<0.05 versus H_2_O_2_. (**c**) Western blot showing the effect of BX-795 on p16 and cyclin A expression after H_2_O_2_ (100 *μ*M) stimulation 60 min in HEK293. (**d**) Statistical analysis of the data shown in (**c**). The data are expressed as the means±s.e.m. from three independent experiments, *P<0.05 versus control, ^&^*P*<0.05 versus H_2_O_2_. (**e**) Representative *β*-gal staining images showing the effect of BX-795 on cell senescence after LPS (1 *μ*g/ml) stimulation 90 min in HEK293. (**f**) Statistical analysis of the data shown in (**e**).The data are expressed as the means±s.e.m. from three independent experiments, *P<0.05 versus control, ^&^*P*<0.05 versus LPS. (**g**) Western blot showing the effect of BX-795 on p16 and cyclin A expression after LPS (1 *μ*g/ml) stimulation 90 min in HEK293. (**h**) Statistical analysis of the data shown in (**g**). The data are expressed as the means±s.e.m. from three independent experiments, *P<0.05 versus control, ^&^*P*<0.05 versus LPS. (**i**) Representative *β*-gal staining images showing the effect of BX-795 on cell senescence after starvation in HEK293. (**j**) Statistical analysis of the data shown in (**i**).The data are expressed as the means±s.e.m. from three independent experiments, *P<0.05 versus control, ^&^*P*<0.05 versus starvation. (**k**) Western blot showing the effect of BX-795 on p16 and cyclin A expression after starvation stimulation. (**l**) Statistical analysis of the data shown in (**k**). The data are expressed as the means±s.e.m. from three independent experiments, *P<0.05 versus control, ^&^*P*<0.05 versus starvation

To investigate the effect of BX-795 on cell senescence and p16 expression *in vitro*, BX-795 with concentration of 6, 60 and 100 nM was injected into HEK293 cells 30 min before stimulation with H_2_O_2_ (100 *μ*M), serum-free culture or 1 *μ*g/ml LPS. The percentage of SA-*β*-gal-staining positive cells induced by H_2_O_2_ was dramatically reduced by BX-795 at a concentration of 6 nM (Figures 4a and b). Although the expression of TBK1 did not differ between cells stimulated with H_2_O_2_ and those treated with H_2_O_2_+BX-795, protein levels of p16 and cyclin A were significantly reduced under H_2_O_2_+BX-795 (6 nM) (Figures 4c and d). In addition, LPS-induced senescence of HEK293 cells decreased following 6-nM BX-795 (Figures 4e and f). Again, TBK1 expression did not differ between cells treated with LPS and those treated with LPS+BX-795, while protein levels of p16 and cyclin A decreased following 6-nM BX-795 (Figures 4g and h). Furthermore, induction of HEK293 cell senescence by serum-free culture (starvation) was reduced by 6 nM BX-795 (Figures 4i and j). Consistent with results from HEK 293 cells stimulated H_2_O_2_ and LPS, western blotting analysis demonstrated that TBK1 expression was not affected by BX-795, while p16 and cyclin A expression levels were significantly reduced after using 6-nM BX-795 (Figures 4k and l). These results indicate that BX-795 at concentration of 6 nM reduces cellular senescence and p16 expression by inhibiting TBK1 activation.

### TBK1-induced upregulation of p16 expression and cell senescence is not p53-dependent

**Figure 5:**
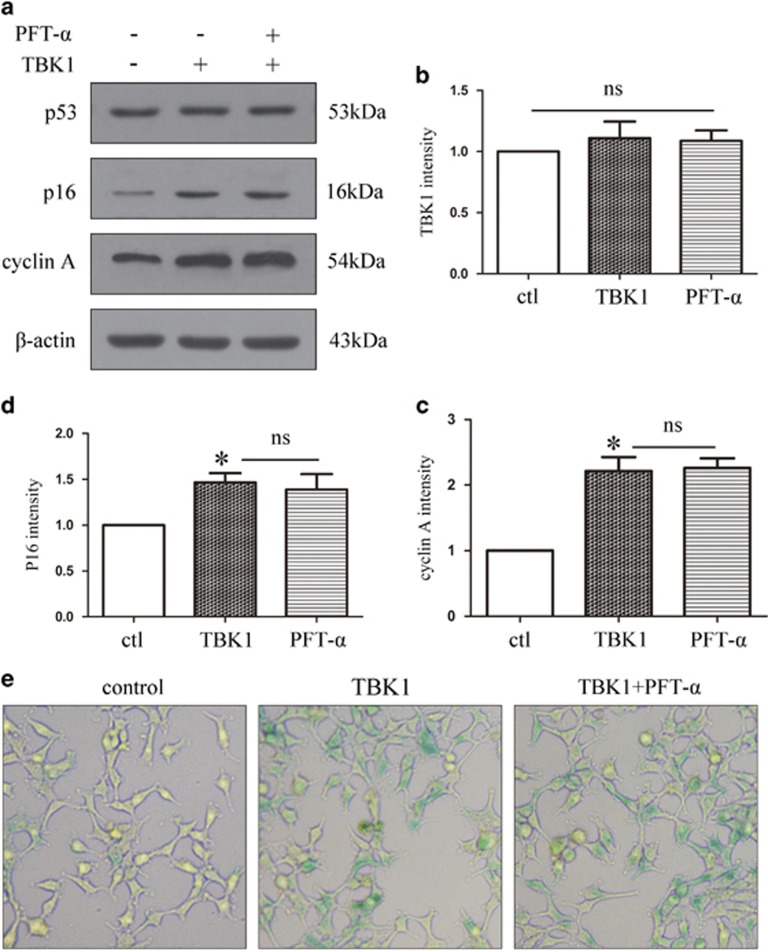
The effect of PFT-α on p16 expression and cell senescence after TBK1 overexpression. (**a**) Western blot showing the effect of p53 inhibitor PFT-α (20 *μ*M) on p16 and cyclin A expression after TBK1 overexpression in HEK293. (**b**-**d**) Statistical analysis of the data shown in (**a**).The data are expressed as the means±s.e.m. from three independent experiments, *P<0.05 versus control. (**e**) Representative *β*-gal staining images showing the effect of PFT-α on cell senescence after TBK1 overexpression in HEK293

To investigate the effect of p53 on p16 expression, we pre-treated HEK293 cells with the p53 inhibitor pifithrin-α (PFT-α) and assessed the effect on p16 expression. First, we found that TBK1 overexpression increased p16 expression but did not affect p53 expression. Furthermore, in cells overexpressing TBK1, pre-treatment with PFT-α did not affect the upregulation of p16 and cyclin A (Figures 5a–d). In addition, SA-*β*-gal staining revealed that PFT-α treatment did not reduce TBK1-induced cell senescence (Figure 5e). These data indicate that TBK1-induced p16 upregulation is not dependent on p53.

### TBK1 increases p16 expression via the Akt-Bmi1 phosphorylation pathway

**Figure 6:**
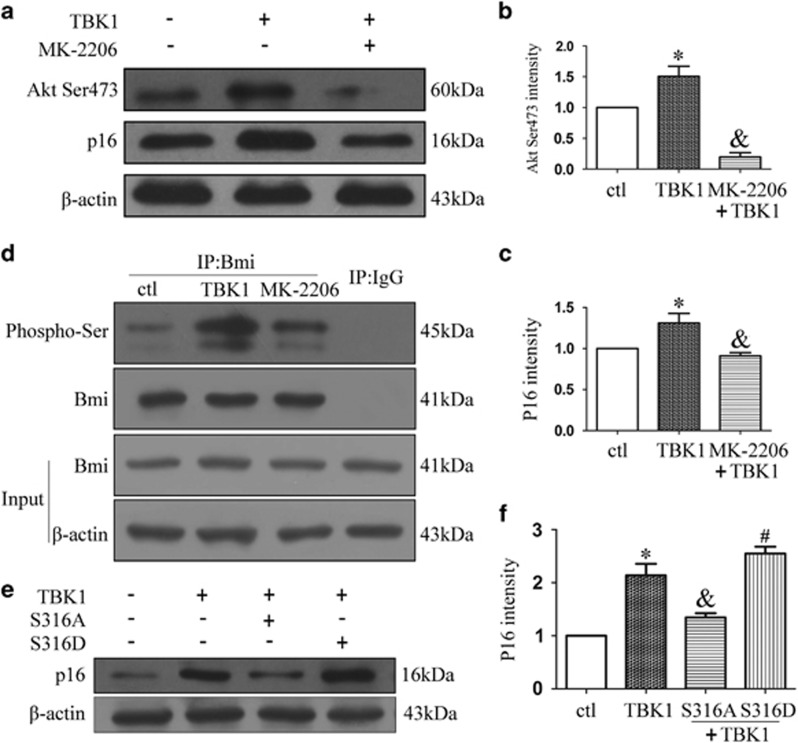
The effect of TBK1 overexpression on Akt-Bmi pathway. (**a**) Western blot showing the effect of MK-2206 (1 *μ*M) on Akt Ser473 and p16 expression after TBK1 overexpression in HEK293. (**b, c**) Statistical analysis of the data shown in (**a**).The data are expressed as the means±s.e.m. from three independent experiments, *P<0.05 versus control, ^&^*P*<0.05 versus TBK1. (**d**) Representative Co-IP results show the interactions of Bmi and phosphorylated serine after TBK1 overexpression in HEK293. (**e**) Western blot showing the effect of Bmi Ser316 mutation on p16 expression after TBK1 overexpression in HEK293. (**f**) Statistical analysis of the data shown in (**e**).The data are expressed as the means±s.e.m. from three independent experiments, *P<0.05 versus control, ^&^*P*<0.05 versus TBK1, ^#^*P*<0.05 versus TBK1+S316A

To determine the mechanism by which TBK1 increases p16 expression, we investigated the Akt-Bmi1 phosphorylation pathway. As shown in Figures 6a–c, western blot results revealed that treatment with MK-2206(1 *μ*M), an Akt inhibitor, decreased the expression of Akt Ser473 in TBK1 hnRNA transfected HEK293 cells, as well as limiting the TBK1-induced upregulation of p16. Co-IP results demonstrated that MK-2206 reduced Bmi1 serine phosphorylation, which increased following TBK1 overexpression (Figure 6d). Furthermore, we found that the TBK1-induced upregulation of p16 could be reversed by a mutation in Bmi1 Ser316 (Figures 6e and f). Taken together, these data indicate that TBK1 increases p16 expression via the Akt Ser473 and Bmi1 Ser316 phosphorylation pathways.

### *TBK1* shRNA knockdown decreases p16 expression and cell senescence *in vitro*

**Figure 7:**
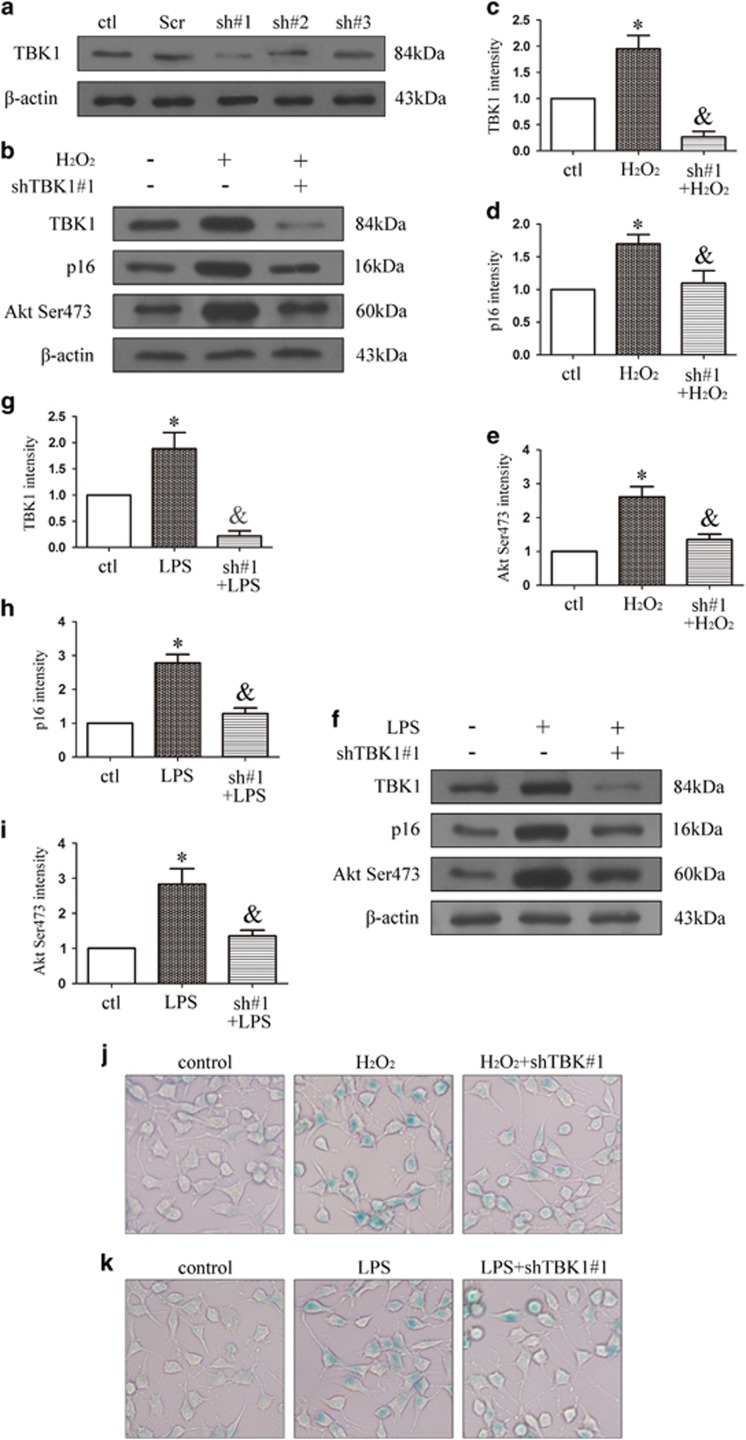
The effect of *TBK1* shRNA on p16 expression and cell senescence *in vitro*. (**a**) Western blot showing the effect of *TBK1* shRNAs on TBK1 expression in N2a. (**b**) Western blot showing the effect of *TBK1* shRNA#1 on p16 and Akt Ser473 expression after H_2_O_2_ (100 *μ*M) stimulation 60 min in N2a. (**c**-**e**) Statistical analysis of the data shown in (**b**).The data are expressed as the means±s.e.m. from three independent experiments, *P<0.05 versus control, ^&^*P*<0.05 versus H_2_O_2_. (**f**) Western blot showing the effect of *TBK1* shRNA#1 on p16 and Akt Ser473 expression after LPS (1 *μ*g/ml) stimulation 90 min in N2a. (**g**-**i**) Statistical analysis of the data shown in (**f**).The data are expressed as the means±s.e.m. from three independent experiments, *P<0.05 versus control, ^&^*P*<0.05 versus LPS. (**j**-**k**) Representative *β*-gal staining images showing the effect of TBK1 shRNA#1 on N2a cell senescence

We designed three *TBK1* shRNAs and assessed knockdown efficiency of TBK1 (Figure 7a and Supplementary Figure 1), the TBK1 shRNA#1 efficiency was remarkable. As shown in Figures 7b–e, *TBK1* shRNA#1 knockdown reduced the expressions of TBK1, p16 and Akt Ser473 following induction of N2a cells by H_2_O_2_. Similar trends were observed following LPS stimulation (Figures 7f–i). SA-*β*-gal staining results revealed that *TBK1* shRNA#1 knockdown decreased N2a senescence under H_2_O_2_ and LPS treatment (Figures 7j and k).

### *TBK1* shRNA knockdown decreases p16 expression and RGC senescence *in vivo*

**Figure 8:**
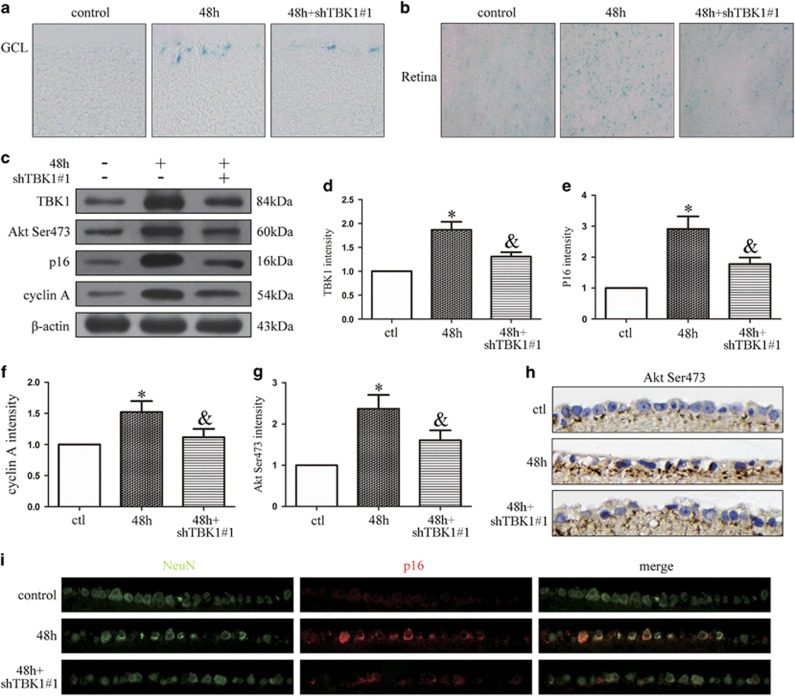
The effect of *TBK1* shRNA on p16 expression and cell senescence *in vivo*. (**a**) Representative *β*-gal staining images of retina slice after acute IOP elevation-induced ischemic injury. (**b**) Representative *β*-gal staining images of retina flat after IOP elevation-induced ischemic injury. (**c**) Western blot showing the effect of *TBK1* shRNA#1 on TBK1, Akt Ser473, p16 and cyclin A expression in ischemic retina. (**d**-**g**) Statistical analysis of the data shown in (**c**).The data are expressed as the means±s.e.m. from three independent experiments, *P<0.05 versus control, ^&^*P*<0.05 versus 48 h. (**h**) Representative immunohistochemistry images show the effect of *TBK1* shRNA#1 on Akt Ser473 expression. (**i**) Representative immunofluorescence images show the effect of TBK1 shRNA#1 on p16 expression

As shown in Figures 8a and b, we injected *TBK1* shRNA#1 into mouse vitreous chambers 10 days before IOP elevation-induced ischemia. SA-*β*-gal staining results demonstrated that *TBK1* shRNA#1 knockdown reduced cell senescence in retinal slices and flats. Western blot results suggested that *TBK1* shRNA#1 knockdown decreased the expressions of TBK1, Akt Ser473, p16 and cyclin A in retinal extracts (Figures 8c–g). Immunohistochemical results demonstrated that *TBK1* shRNA#1 knockdown acted to downregulate Akt Ser473 expression in the RGC layer (Figure 8h). Immunofluorescence results indicated that *TBK1* shRNA#1 knockdown decreased the expression of p16 in RGCs (Figure 8i).

## Discussion

Glaucoma is the leading cause of blindness, and elevated IOP is an important risk factor for RGC death in retinal ischemia and glaucoma;^3, 20, 21^ however, the relationship between high IOP and RGC death is not completely understood. Cell senescence is a state of irreversible growth arrest, and increased p16 expression in RGCs can cause RGC death and subsequent blindness. In the present study, we investigated the mechanism by which acute elevated IOP-induced ischemic injury upregulates TBK1 and p16 expression in the ganglion cell layer. We found that an elevated IOP triggers increased expression of TBK1, directly activating Akt Ser473 phosphorylation in cells, impairing Bmi1 function by increasing Bmi1 serine phosphorylation, and inducing p16 expression in RGCs following ischemic retinal injury. Consistent with this model, silencing of *TBK1* via shRNA knockdown or the TBK1 inhibitor BX-795 significantly attenuated p16 expression in RGCs as well as reducing SA-*β*-gal staining in the ganglion cell layer after IOP-induced retinal ischemic damage.

Typically, IKK-related kinases TBK1 are involved in the activation of various cellular pathways leading to interferon and pro-inflammatory cytokine production as well as homeostatic cellular functions such as cell renewal and proliferation. As a potent and relatively specific inhibitor of TBK1, BX-795 blocked the phosphorylation, nuclear translocation, and was used for further research of TBK1.^8, 22^ Recently, TBK1 has been found to modulate autophagy through its catalytic activity, inducing death in retinal cells;^6^ this provides the molecular link between TBK1 and RGC death. Moreover, cellular senescence plays a critical role in the pathogenesis of glaucoma, and the p16 upregulation triggered by acute IOP elevation appears to be a cause of RGC death.^3, 23^ Our findings demonstrate that upregulation of TBK1 and p16 is associated with elevated IOP and that elimination of TBK1 diminishes p16 and cyclin A expression. In addition, TBK1 overexpression could not be mitigated by treatment with the p53 inhibitor PFT-α. These findings indicate that TBK1 induces the upregulation of p16 during the development of acute elevated IOP-induced RGC senescence.

Bmi1 is primarily considered a potent inhibitor of the Ink4a-Arf locus; p16 and p19Arf act as critical downstream targets for Bmi1 with regard to its effects on cell proliferation and senescence. Bmi1-deficient mice exhibit neurological disorders, demonstrating the essential role of Bmi1 in regulating the self-renewal of neural stem cells.^14, 24, 25^ Site-specific phosphorylation of Bmi1 by various kinases influences its tumorigenicity, both positively and negatively, and the phosphorylation of Bmi1 at different serine residues modulates its function through the Ink/Arf pathway.^26^ Our findings demonstrate that inhibition of Akt Ser473 phosphorylation decreases Bmi1 phosphorylation. Moreover, TBK1-induced upregulation of p16 is rescued by the Bmi1 S316A mutant but not by the Bmi1 S316D mutant, indicating that Bmi1 Ser316 phosphorylation is an important link between Akt and p16 in IOP-induced RGC senescence. Consistent with our result, Liu *et al* also observed that Akt-mediated phosphorylation of Bmi1 Ser316 leads to its dissociation from chromatin and to derepression of the Ink4a-Arf locus.^16^

Although Bmi1 is a direct substrate for Akt, the PI3K/Akt pathway stimulates phosphorylation of Bmi1 Ser316 and inhibits its function. Some reports have found that Bmi1 phosphorylation is positively modulated by pAkt levels. Additionally, RNAi-mediated *TBK1* depletion, and pharmacological inhibition of TBK1 kinase activity, reveals TBK1 as a targetable link supporting context-selective mobilization of the Akt regulatory network.^17, 27^ The present study demonstrates that inhibition of Akt Ser473 phosphorylation suppresses Bmi1 phosphorylation and TBK1-induced upregulation of p16. This p16 upregulation was partially blocked by Bmi1 S316A phosphorylation but not by Bmi1 S316D phosphorylation. Meanwhile, inhibition of TBK1 decreased the activation of Akt Ser473 phosphorylation and the expression of p16. These data indicate that TBK1 upregulation induces Akt Ser473 phosphorylation, Bmi1Ser316 phosphorylation and p16 expression, thereby inducing cell senescence.

**Figure 9:**
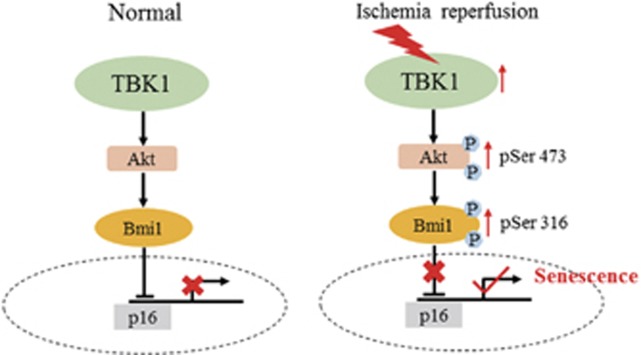
Schematic representation of the contribution of TBK1 to RGC senescence after acute IOP elevation-induced ischemic retinas. Acute IOP elevation-induced ischemic retinas induces TBK1 up-regulation, which then increases Akt Ser473 and Bmi Ser316 phosphorylation, whereby upregulates p16 expression, finally induces RGC senescence

Cellular senescence plays a critical role in the pathogenesis of glaucoma. RGC senescence can result in RGC death and subsequent blindness. Although some reports have found that TBK1-induced cell death of RGC-5 cells is dependent on autophagy,^6^ Akt connects TBK1 to pro-survival signalling.^17^ Akt also phosphorylates Bmi1 to block the Ink4a-Arf locus.^16, 18^ The molecular mechanism of TBK1-induced RGC senescence in elevated IOP-induced ischemic retinas therefore remains elusive. In the current study, we observed that the expression of TBK1 and RGC senescence peaked at 48 h after acute elevated IOP-induced retinal ischemia. IOP elevation significantly increased TBK1 expression and RGC senescence, but these could be mitigated by treatment with *TBK1* shRNA, BX-795,^22^ or MK-2206.^28^ Notably, our findings provide a molecular link and pathway between TBK1 and p16, with TBK1 upregulation directly activating Akt Ser473 phosphorylation, Bmi1 Ser316 phosphorylation, p16 expression and RGC senescence in the pathogenesis of elevated IOP-induced retinal ischemia (Figure 9). On the basis of these observations, our results provide important insight into the pathogenesis of acute IOP-induced retinal ischemia. Moreover, our findings indicate that suppression of the TBK1/Akt Ser473/Bmi1 Ser316/p16 axis is a potential therapeutic strategy for minimizing RGC senescence in retinal ischemia and glaucoma.

## Materials and methods

### Animals

Male, 3-month-old C57BL/6J mice (20–25 g in weight, The Experiment Animal Center of the Tongji Medical College, HUST, China) were housed in covered cages, fed with a standard rodent diet ad libitum and kept on a 12-h light/12-h dark cycle. All procedures concerning animals were performed in accordance with the ARVO statement for the Use of Animals in Ophthalmic and Vision Research and under protocols approved by institutional IACUC committees at Huazhong University of Science and Technology.

### Ischemia reperfusion mouse model

The mice were anesthetized by intraperitoneal injections of 10 ml/kg of 4% chloral hydrate. The corneas were topically anesthetized with 0.5% tetracaine hydrochloride, and the pupils were dilated with 1% tropicamide. A 30-gauge needle was inserted into the anterior chamber of right eye that was connected by flexible tubing to a saline reservoir. By raising the reservoir, IOP was elevated and maintained to 75 mm Hg for 45 min.^3, 20, 21^ Retinal ischemia was confirmed by whitening of the iris and loss of the red reflex, and subsequent reperfusion was evident by the return of the red reflex. The left eye served as a control. After 45 min, the needle was withdrawn and tobramycin was applied to avoid bacterial infection. Mice were killed at 6, 24, 48 or 72 h after the procedure.

### Reagents and antibodies

PFT-α was obtained from Sigma-Aldrich China (Shanghai, China; P4236). The TBK inhibitor BX-795 and the Akt inhibitor MK-2206 were purchased from Selleck chemicals (Houston, TX, USA) (s1274 and s1078). Senescence *β*-gal staining kit (Cell Signaling Technology, Beverly, MA, USA; #9860).

The following antibodies were used in western blot: anti-TBK1 (Abcam, Cambridge, MA, USA, ab-40676, 1:1000), anti-p16 (Abcam, ab-51243, 1:1000), and anti-cyclin A (Santa Cruz, Dallas, TX, USA; sc-596, 1:1000), anti-pAkt 473 (Cell Signaling Technology; #4060, 1:2000), anti-Bmi (Cell Signaling Technology; #648, 1:1000), anti-phosphorylation serine (Boster, China, BM1622), anti-p53 (Santa Cruz; sc-126, 1:1000), anti-*β*-actin (Santa Cruz; sc-47778, 1:1000). The following antibodies were used in immunofluorescence: anti-NeuN (Abcam, ab-177487, 1:200), anti-Brn-3a (Santa Cruz; sc-8429, 1:200), anti-p16 (Abcam, ab-51243, 1:100), anti-TBK1 (Abcam, ab-40676, 1:200). Anti-pAkt 473 (Cell Signaling Technology; #4060, 1:200) was used in immunohistochemistry.

### Intravitreal injections

The experimental eyes were injected with the TBK1 inhibitor BX-795 (50 nM/2 *μ*l, 500 nM/2 *μ*l) or TBK1 shRNA into the vitreous cavity through a 35-G needle with a 10-*μ*l Hamilton microsyringe (Hamilton, Reno, NV, USA) before the onset of reperfusion. Tobramycin was applied to prevent bacterial infection.

### Frozen sections

At the experimental time points, eyes were enucleated and fixed with 4% paraformaldehyde overnight and dehydrated with 20% sucrose overnight prior to embedding in optimum cutting temperature compound (Tissue-Tek, Sakura Finetek Inc, Tokyo, Japan). Embedded eyes were directly frozen in a cryostat (Leica CM1900, Berlin, Germany) at −20 °C. Six-*μ*m serial frozen sections were cut along the vertical meridian, air-dried, and stained with SA-*β*-gal staining.

### Retinal flat and RGCs quantification

At the experimental time points, mice were killed with CO_2_, and eyeballs were dissected and fixed in ice-cold phosphate-buffered 4% paraformaldehyde (pH=7.4) for 30 min, followed by flat mounting of the retinas. To detect senescent RGCs, flat mounts were incubated overnight at 37 °C with an SA-*β*-gal staining kit (Cell Signaling). × 400 images were obtained from the same quadrant of retinal flat mounts and analysed with a confocal microscope (Carl Zeiss, Inc, Germany).

### Senescence-associated *β*-galactosidase staining (SA-*β*-gal)

Senescence assays were performed using the Senescence *β*-galactosidase staining kit (Cell Signaling) according to the manufacturer's protocol. Senescent RGCs, HEK293 cells and N2a cells expressing SA-*β*-gal were stained blue.

### Immunofluorescence

The paraffin-embedded retinal sections (5 *μ*m) were gently washed twice with PBS pre-heated to 37 °C. Afterwards, the sections were treated again with 5% bovine serum albumin for the additional 60 min to block non-specific binding, and incubated with rabbit polyclonal TBK1 antibody, rabbit polyclonal p16 antibody, mouse monoclonal Brn-3a antibody, or mouse monoclonal NeuN antibody in 5% bovine serum albumin at 4 °C overnight. Retinal paraffin sections were washed and incubated with the conjugated secondary antibody (1:200; Invitrogen) in 5% bovine serum albumin (37 °C, 1 h). Double-photon fluorescence microscope (Zeiss 510 Meta, Zeiss, Germany) was used to detect the fluorescence. Enhanced green fluorescent protein and rhodamine were excited using light at 488 and 543 nm, respectively.

### Cell culture

HEK293 cells and N2a cells were maintained in DMEM supplemented with 10% FBS (Gibco, CA, USA), penicillin (100 U/ml)/streptomycin (100 *μ*g/ml) and 2 mM L-glutamine at 37 °C in a 5% CO_2_. Confluent cell layers were split two times per week.

### Plasmid construct and TBK1 shRNA

DNA fragments corresponding to the full-length *TBK1* were amplified by PCR, followed by cloning into the pEGFP-N1 vector (Invitrogen, Carlsbad, CA, USA). Mouse *TBK1* shRNA plasmids were purchased from GeneChem (Shanghai, China). Plasmid encoding the *Bmi* fragment were constructed according to standard protocol. The Ser316 mutation to Ala (SA) or Asp (SD) was achieved using the Fast Mutagenesis System (TRANSGEN, Beijing, China). HEK293 and N_2_A cells were then transfected using Lipofectamine 2000 reagents (Invitrogen).

The shRNA sequence that targets mouse *TBK1* sequence (GenBank No. NM_019786) was designed as follows: 5*′-GTTTAAAGATAAGTCGGAA′-*3.

### Co-IP

Briefly, cell lysates were generated by sonication in a buffer containing 20 mM HEPES, 400 mM KCl, 5% glycerol, 5 mM EDTA, 0.4% NP-40 and protease inhibitors, and precleared by centrifugation. The cell lysates were then incubated with an anti-Bmi antibody overnight at 4 °C. The reaction mixture was then incubated with protein A/G PLUS-Agarose beads (Santa Cruz; sc-2003) for 2 h at 4 °C. The precipitates were washed three times with wash buffer and then eluted from the protein A/G PLUS-Agarose beads by boiling with 1X sodium dodecyl sulfate (SDS) for 5 min at 95 °C. The protein samples were resolved by SDS-polyacrylamide gel electrophoresis.

### Western blot analysis

For analysis of protein expression, total protein was isolated and harvested from retina sample, the cultured HEK293 cells and N_2_A cells at designed time-points. Samples were separated using 10, 12 or 15% polyacrylamide gels and transferred to polyvinylidene difluoride membranes. Polyvinylidene difluoride membranes were blocked with 5% bovine serum albumin at room temperature for 60–90 min, and afterwards incubated overnight at 4 °C with antigen-specific primary antibodies. Blots were incubated with species-specific horseradish peroxidase-conjugated secondary antibodies for 60 min at room temperature. Proteins were visualized using chemiluminescence substrate kit (ECL Plus; PerkinElmer Inc, Covina, CA, USA). Target proteins were quantified using ImageJ software after normalizing the expression levels to *β*-actin level.

### Statistical analysis

Data are expressed as means±s.e.m. One-way ANOVA, followed by the Dunnett's multiple comparison tests and two-way ANOVA were performed using GraphPad Prism software (version 5.0, GraphPad Software). All statistical assessments were two-sided, and *P* values less than 0.05 were considered statistically significant.

## Supplementary Material

Supplmentary Information

Supplmentary Figure 1
