# Supplementary material for: P16INK4a upregulation mediated by TBK1 induces retinal ganglion cell senescence in ischemic injury
Source: Cell Death Dis. 2017 Apr 20;8(4):e2752–. doi: 10.1038/cddis.2017.169 (PMC5477587; doi:10.1038/cddis.2017.169)
Supplement: Supplmentary Information [file cddis2017169x1.docx]

**Supplementary Information for:**

**P16****INK4a upregulation mediated by TBK1 induces retinal ganglion cell senescence in ischemic injury**

Lu Li^1,2,3^, Yin Zhao^1,3^, Hong Zhang^1*^

^1^Department of Ophthalmology, Tongji Hospital, Tongji Medical College, Huazhong University of Science and Technology, Wuhan 430030, China.

^2^Department of Ophthalmology, The First Affiliated Hospital, Shihezi University School of Medicine, Shihezi, 832002 Xinjiang, People's Republic of China.

^3^These authors contributed equally to this work.

**Supplementary figure legends:**

**Supplementary Figure S1:** TBK1 reduced by shTBK#1 in vivo. (a) representative immunohistochemistry result shows the effect of shTBK1#1 on TBK1 expression in retina slice. (b) representative immunofluorescence result shows the effect of shTBK1#1 on TBK1 expression in retina slice.
