# Supplementary figures and images for: P16INK4a upregulation mediated by TBK1 induces retinal ganglion cell senescence in ischemic injury
Source: Cell Death Dis. 2017 Apr 20;8(4):e2752–. doi: 10.1038/cddis.2017.169 (PMC5477587; doi:10.1038/cddis.2017.169)

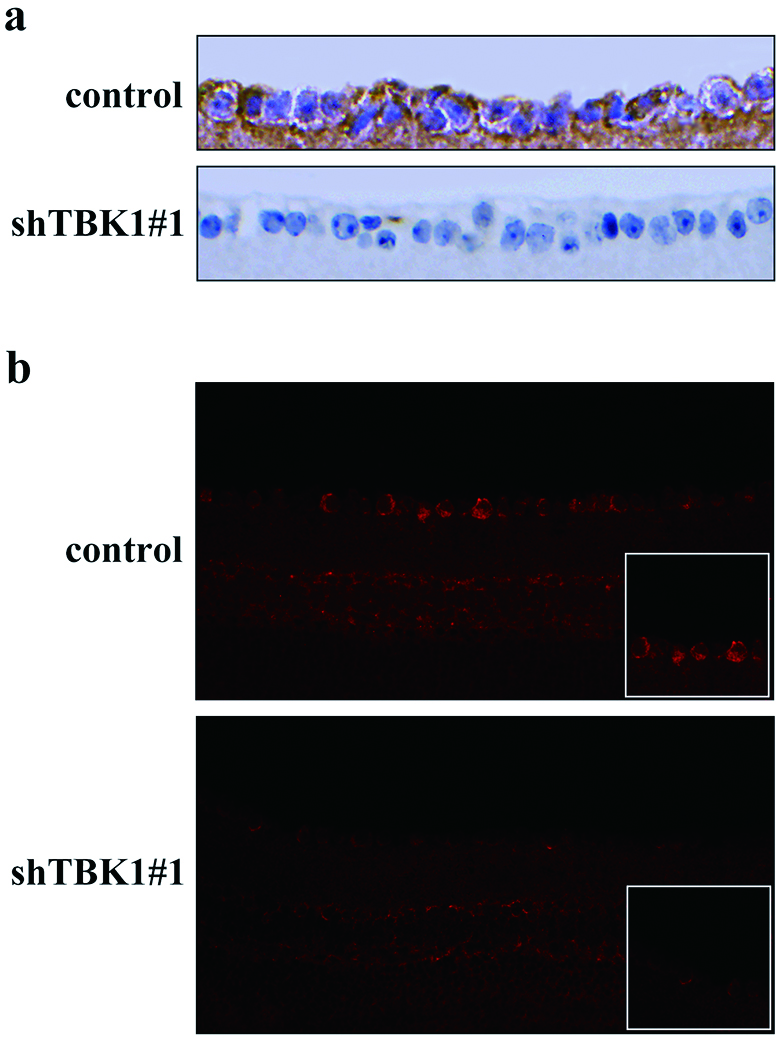

Supplement: Supplmentary Figure 1 [file cddis2017169x2.tif]
